# Molecular epidemiology and outcome of carbapenem-resistant Enterobacterales in Saudi Arabia

**DOI:** 10.1186/s12879-022-07507-y

**Published:** 2022-06-13

**Authors:** Basem M. Alraddadi, Emily L. G. Heaphy, Yamama Aljishi, Waleed Ahmed, Khalid Eljaaly, Hanan H. Al-Turkistani, Abeer N. Alshukairi, Mohammed O. Qutub, Kholoud Alodini, Roaa Alosaimi, Waseem Hassan, Dalya Attalah, Rakan Alswaiel, Mohammed F. Saeedi, Mohammed A. Al-Hamzi, Lama K. Hefni, Reem S. Almaghrabi, Mushira Anani, Abdulhakeem Althaqafi

**Affiliations:** 1 King Faisal Specialist Hospital and Research Center, Jeddah, Saudi Arabia; 2 Alfaisal University, Riyadh, Saudi Arabia; 3 King Fahd Specialist Hospital, Dammam, Saudi Arabia; 4 Security Forces Hospital, Makkah, Saudi Arabia; 5 Faculty of Pharmacy, King Abdulaziz University, Jeddah, Saudi Arabia; 6 King Abdullah Medical City, Makkah, Saudi Arabia; 7 Department of Pathology and Laboratory Medicine Clinical Microbiology Lab, King Faisal Specialist Hospital and Research Center, Jeddah, Saudi Arabia; 8 Department of Medicine, King Abdulaziz Medical City, Jeddah, Saudi Arabia; 9 Clinical Microbiology Laboratory, King Abdulaziz University Hospital, Jeddah, Saudi Arabia; 10 King Fahd Medical City, Riyadh, Saudi Arabia; 11 King Faisal Specialist Hospital and Research Center, Riyadh, Saudi Arabia; 12 Present Address: King Saud Bin Abdulaziz University for Health Sciences, Jeddah, Saudi Arabia

**Keywords:** Carbapenem-resistant Enterobacterales (CRE), Saudi Arabia, OXA-48, Multidrug resistant gram negative bacteria

## Abstract

**Background:**

The burden of carbapenem resistance is not well studied in the Middle East. We aimed to describe the molecular epidemiology and outcome of carbapenem-resistant Enterobacterales (CRE) infections from several Saudi Arabian Centers.

**Methods:**

This is a multicenter prospective cohort study conducted over a 28-month period. Patients older than 14 years of age with a positive CRE *Escherichia coli* or *Klebsiella pneumoniae* culture and a clinically established infection were included in this study. Univariate and multivariable logistic models were constructed to assess the relationship between the outcome of 30-day all-cause mortality and possible continuous and categorical predictor variables.

**Results:**

A total of 189 patients were included. The median patient age was 62.8 years and 54.0% were male. The most common CRE infections were nosocomial pneumonia (23.8%) and complicated urinary tract infection (23.8%) and 77 patients (40.7%) had CRE bacteremia. OXA-48 was the most prevalent gene (69.3%). While 100 patients (52.9%) had a clinical cure, 57 patients (30.2%) had died within 30 days and 23 patients (12.2%) relapsed. Univariate analysis to predict 30-day mortality revealed that the following variables are associated with mortality: older age, high Charlson comorbidity index, increased Pitt bacteremia score, nosocomial pneumonia, CRE bacteremia and diabetes mellitus. In multivariable analysis, CRE bacteremia remained as an independent predictor of 30 day all-cause mortality [AOR and 95% CI = 2.81(1.26–6.24), p = 0.01].

**Conclusions:**

These data highlight the molecular epidemiology and outcomes of CRE infection in Saudi Arabia and will inform future studies to address preventive and management interventions.

## Introduction

The emergence of carbapenem-resistant Enterobacterales (CRE) infections represents a significant public health threat [1, 2]. The World Health Organization (WHO) considers CRE infection a priority that needs to be addressed globally [3]. Carbapenems are considered the treatment of choice for extended-spectrum β-lactamase bacteria and the last line of defense against Enterobacterales. CRE infections are associated with poor outcomes due to limited treatment options and inappropriate initial therapy [4–6]. Evidence also suggests that infections with CRE are associated with worse outcomes when compared with susceptible Enterobacterales [4, 5, 7, 8]. Furthermore, CRE infection is associated with longer hospital stays and higher healthcare costs [5, 7–9].

Knoweldge regarding the molecular epidemiology of CRE in Gulf countries and in the Middle East in general is limited. In Saudi Arabia, studies have shown that the most prevalent Carbapenemase strain is OXA-48 followed by NDM [10, 11]. Most of these studies are limited by small sample sizes and retrospective designs. Saudi’s wide geographic area and the continuous influx of international and domestic pilgrims are likely factors affecting CRE strain distribution. The exact molecular epidemiology and impact of CRE on outcomes are still not clear. Treatment options for CRE infection remain inadequate. Available options are limited by pharmacokinetics challenges, toxicity, and availability. Understanding the molecular epidemiology of CRE in the region and the impact it has on health care is needed to inform local guidelines.

Our aim in this study is to describe the molecular epidemiology, clinical features, and outcomes of patients infected with CRE from several hospitals in Saudi Arabia. We also aimed to identify predictors of mortality in this population.

## Methods

Patients in this multicenter prospective cohort study were recruited over a 28-month period from August 2018 through November 2020 in eight hospital systems in the Kingdom of Saudi Arabia. Hospitals with a median of 462.5 beds in the cities of Jeddah, Makkah, Dammam, and Riyadh participated in enrolling patients. Patients were excluded from participating in the study if they were 14 years of age or less. The final study population consisted of individuals above the age of 14 years with a positive CRE *Escherichia coli* or *Klebsiella pneumoniae* culture and a clinically established infection.

Susceptibility testing was performed using the Vitek 2 system (bioMérieux, Marcy L’étoile, France) and N-291 card. Phenotypic confirmation of CRE was performed using the Clinical Laboratory Standards Institute (CLSI) methodology, which includes the ertapenem, imipenem, and meropenem E-test and modified Hodge test (MHT). All confirmed isolates of CRE from the culture were then tested using the Xpert Carba-R Kit following the manufacturer’s recommendation for rapid detection and differentiation of the *bla*_KPC_, *bla*_NDM_, *bla*_VIM_, *bla*_OXA-48_, and *bla*_IMP_ gene sequences linked to carbapenem resistance in gram-negative bacteria. The Xpert Carba-R Assay will generate a negative or non-Detected result when testing samples resistance to Carbapenem but containing other than *bla*_KPC_, *bla*_NDM_, *bla*_VIM_, *bla*_OXA-48_, and *bla*_IMP_ gene sequences. The interpretation of the minimum inhibitory concentration (MIC) for carbapenems is based on CLSI guidelines; resistance to ertapenem was considered if MIC was ≥ 2 μg/ml and resistance to meropenem and imipenem was considered if MICs were ≥ 4 μg/ml.

The primary outcome in this study was 30-day all-cause mortality. Length of hospital stay, attributable mortality, relapse within 30 days, and acute kidney injury were recorded. Demographic and clinical characteristics including age, gender, comorbidities, Charlson Comorbidity Index, Apache II score, Pitt bacteremia score, type of CRE infection, organism isolates, location in the hospital at the time of CRE culture, molecular typing, and type of treatment patients received were considered as possible exposures and predictors of 30 day all-cause mortality. Susceptibility to and minimum inhibitory concentrations (MICs) for meropenem, imipenem, ertapenem, and ceftazidime-avibactam (CAZ-AVI) treatments were obtained for the CRE bacteria isolates. Normality of the continuous variables was assessed using Shapiro–Wilk and Kolmogorov–Smirnov tests and by comparing median and mean values.

The associations between 30 day all-cause mortality and continuous and categorical predictors were described using the Kruskal–Wallis and Chi-Square tests. Univariate logistic models were also constructed to assess the relationship between the outcome of 30 day all-cause mortality and possible continuous and categorical predictor variables. All predictor variables with a p-value < 0.1 in univariate analysis models were included in a multivariable logistic model to predict 30 day all-cause mortality. A similar analysis was conducted for the group of patients who were bacteremic (n = 77). Analyses were performed using SAS software, version 9.4, and statistical significance was determined at an α = 0.05 level. The study was approved by the institutional review boards at all participating hospitals.

## Results

The study population included 189 patients greater than 14 years of age with a positive CRE culture and a clinically established infection. The median patient age was 62.8 years and 54.0% were male (Table 1). Just over one-fifth (20.1%) of the participants had heart disease and 42.3% had diabetes mellitus. Almost one-third (32.8%) of the patients had a non-hematological malignancy and 19.6% had a cerebral vascular accident comorbidity. The median Charlson comorbidity index was six at baseline. The APACHE II median score for patients who required ICU admission was 19 (n = 112) while the Pitt bacteremia median score for patients with bacteremia was two (n = 110) (Table 1).

**Table 1:** Demographic and baseline clinical characteristics of patients with CRE infection (n = 189)

Variable	n (%)	Median (IQR)
Age (years)		62.8 (44.4–73.5)
Gender		
Male	102 (54.0)	
Female	87 (46.0)	
Baseline Comorbidities		
Heart Disease	38 (20.1)	
Peripheral vascular disease	9 (4.8)	
Cerebral vascular accident	37 (19.6)	
Chronic kidney disease	28 (14.8)	
End stage renal disease	10 (5.3)	
HIV	1 (0.5)	
Dementia	19 (10.1)	
COPD	6 (3.2)	
Connective tissue disease	5 (2.7)	
Peptic ulcer disease	7 (3.7)	
Mild liver disease	7 (3.7)	
Moderate and severe liver disease	22 (11.6)	
Diabetes Mellitus	80 (42.3)	
Non-hematological malignancy	62 (32.8)	
Hematological malignancy	11 (5.8)	
Solid organ transplant	8 (4.2)	
Bone marrow transplant	4 (2.1)	
Immunosuppressive medication	41 (21.7)	
Charlson comorbiditiy index		6 (3–8)
APACHE II score^1^		19 (9.0–31.5)
Pitt bacteremia score^2^		2 (1–6)
Type of CRE infection^3^		
CLABSI	28 (14.8)	
HAP/VAP	45 (23.8)	
Complicated UTI	45 (23.8)	
Complicated intra-abdominal infection	32 (16.9)	
Skin and soft tissue infection	20 (10.6)	
Joint/bone infection	6 (3.2)	
Primary bacteremia (source unidentified)	15 (7.9)	
Other	19 (10.1)	
Organism isolates^4^		
* Escherichia coli*	21 (11.1)	
* Klebsiella pneumoniae*	165 (87.3)	
Both *E. coli* and *K. pneumoniae*	2 (1.1)	
CRE bacteremia	77 (40.7)	
Location at time of CRE culture		
Emergency Room	20 (10.6)	
Medical Floor	52 (27.5)	
Surgical Floor	21 (11.1)	
Hematology/Oncology Floor	29 (15.3)	
OB/GYN Floor	1 (0.5)	
ICU	57 (30.2)	
Other	9 (4.8)	
Molecular typing^5^		
KPC	1 (0.5)	
NDM	32 (16.9)	
IMP	1 (0.5)	
OXA_48	131 (69.3)	
None identified	25 (13.2)	

*IQR* interquartile range, *CLABSI* central line-associated blood stream infection, *HAP* hospital-acquired pneumonia, *CRE* Carbapenem-resistant *Enterobacteriaceae*

^1^For n = 112 patients, ^2^For n = 110 patients, ^3^More than one CRE infection type possible

per patient, ^4^The isolates were identified at the species level following conventional schemes, percentages may total < 100 due to missing values, ^5^More than one molecular typing possible per patient

Almost half of the patients had either hospital-acquired pneumonia (HAP) or ventilator-associated pneumonia (VAP) (23.8%) or a complicated urinary tract infection (UTI) (23.8%) caused by CRE (Table 1). The majority of organisms isolated were *Klebsiella pneumoniae* (87.3%). Seventy-seven patients (40.7%) had CRE bacteremia and approximately one-third (30.2%) of patients were in the ICU at the time they received a positive CRE culture (Table 1).

For the isolates tested, the median MIC was greatest for meropenem with a value of 16 followed by imipenem with a value of 8. Of the 143 isolates tested for CAZ-AVI, 103 (72.0%) were susceptible, 35 (24.5%) were resistant, and 5 (3.5%) were intermediate at baseline. For the isolates resistant to CAZ-AVI, the molecular typing was KPC (n = 1), NDM (n = 24), and OXA-48 (n = 15) with some overlap between KPC and NDM being found in 1 patient and NDM and OXA-48 being found in 8 patients. The median MIC was smallest for CAZ-AVI with a value of 2 for the isolates tested against this antibiotic. Molecular typing revealed over two-thirds (69.3%) of patients were infected with OXA-48 CRE (Table 1).

**Fig. 1:**
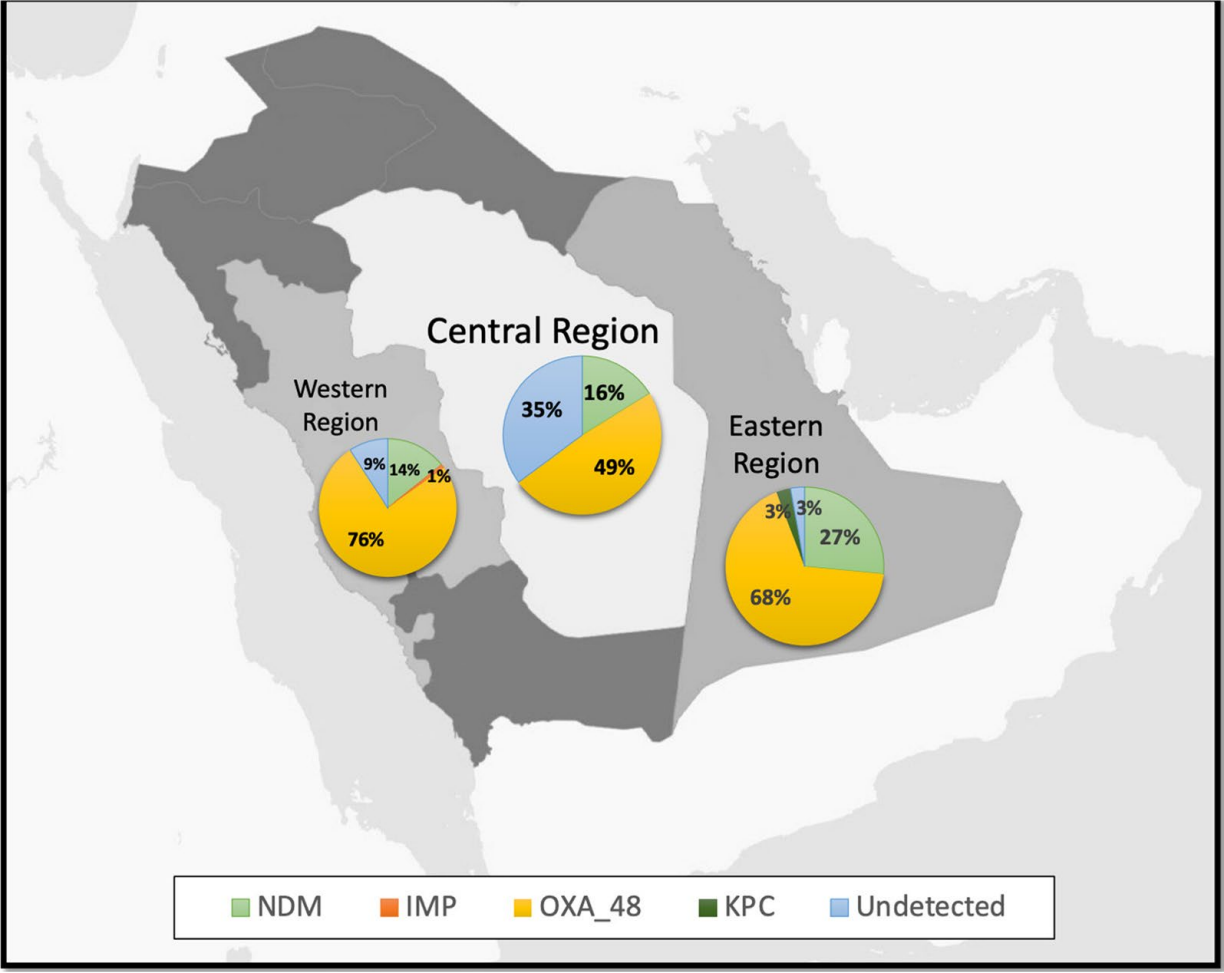
Percentages of CRE molecular typing identified in different regions of Saudi Arabia

Overall, the study included 114 patients from western region hospitals, 41 patients from the central region of the Kingdom, and 34 patients from the Eastern province (Fig. 1). While 78.9% of patients in the western region had OXA_48 molecular typing identified in their isolates, 43.9% of patients in the central region and 67.7% of patients in the eastern region had OXA_48 identified in their isolates. A similar percentage of the patient isolates were identified as NDM in the western (14.9%) and central (14.6%) regions compared to 26.5% of patient isolates being identified as NDM in the eastern region. Almost one-third of isolates (31.7%) from patients in the central region had no molecular typing detected (Fig. 1).

While 69.3% of patients took a carbapenem as part of their treatment regimen, 47.1% of patients received CAZ-AVI (Table 2). Approximately one-fifth (20.1%) of patients received an aminoglycoside at some point during their treatment regimen (Table 2). There were 100 patients (52.9%) with a clinical cure and 57 patients (30.2%) had died within 30 days (Table 3). Over one-third (36.5%) of patients had acute kidney injury and 23 patients (12.2%) relapsed within 30 days (Table 3).

**Table 2:** Treatment regimen for patients with CRE infection

Variable	n (%)
Any carbapenem (meropenem, imipenem, ertapenem)	131 (69.3)
Aminoglycoside (amikacin, gentamicin)	38 (20.1)
Aztreonam	11 (5.8)
CAZ-AVI	89 (47.1)
Colistin	78 (41.3)
Tigecycline	62 (32.8)

*CAZ-AVI* ceftazidime-avibactam

**Table 3:** Outcomes of patients with CRE infections (n = 189)

Variable	n (%)
30 day all-cause mortality	57 (30.2)
Attributable mortality	44 (23.4)
Clinical cure	100 (52.9)
Relapse within 30 days	23 (12.2)
Acute kidney injury	69 (36.5)

The median age of patients who died within 30 days at 64.7 years was significantly different than the median age, 60.3 years, in the group of patients who did not die within 30 days (Kruskal–Wallis Test = 4.8, df = 1, p = 0.03). Similarly, the association of Charlson comorbidity index with 30 day mortality was significant with a median of 6.0 in the group of patients who died within 30 days compared to a median of 5.5 in the group of patients who did not die within 30 days (Kruskal–Wallis Test = 9.1, df = 1, p = 0.003). The median values of Pitt bacteremia were also significantly different between the two groups with a value of 6.0 in the group of patients who died compared to a median value of 1.0 in the group of patients who did not die within 30 days (Kruskal–Wallis Test = 15.3, df = 1, p < 0.0001).

There was a statistically significant difference between the patients who had a HAP/VAP CRE infection with regards to 30 day all-cause mortality (Chi-Square = 5.7, df = 1, p = 0.02). While 35.1% of the patients who died within 30 days had a HAP/VAP infection, 18.9% of patients who did not die within 30 days had a HAP/VAP infection. The association between having CRE bacteremia and 30 day all-cause mortality was also statistically significant with 57.9% of patients who died also having CRE bacteremia compared to 33.3% of patients who did not die having CRE bacteremia (Chi-Square = 9.9, df = 1, p = 0.002). Presenting with the comorbid condition of Diabetes Mellitus was also significantly associated with 30 day all-cause mortality with 54.4% of patients who died having this condition compared to 37.1% of patients who did not die within 30 days having this condition (Chi-Square = 4.9, df = 1, p = 0.03).

There was no significant association identified between CRE treatment with CAZ-AVI and 30 day all-cause mortality (Chi-Square = 0.8, df = 1, p = 0.37). Twenty four (27.0%) of the patients who took CAZ-AVI ended up dying within 30 days of all-cause mortality. Approximately one third (n = 33, 33.0%) of patients who did not take CAZ-AVI (n = 97) ended up dying within 30 days of all-cause mortality.

In further looking at the association of CAZ-AVI treatment with 30 day all-cause mortality, among the patients with isolates that tested sensitive to CAZ-AVI and who took CAZ-AVI as part of their treatment regimen compared to the rest of the patients, there was no significant relationship. The association between those who tested sensitive compared to those with resistant or intermediate sensitivity results to CAZ-AVI for the patients who took CAZ-AVI was also not significant with regards to 30 day all-cause mortality. In addition, taking CAZ-AVI treatment did not significantly predict whether or not patients had a clinical cure [OR and 95% CI = 1.52 (0.86–2.71), p = 0.15]. The outcome of acute kidney injury was not significantly associated with CAZ-AVI treatment [OR and 95% CI 1.15 (0.63–2.08), p = 0.65].

Patients with CRE bacteremia (n = 77) had a median age of 63.6 years, a median Charlson comorbidity index of 6.0, and a median Pitt bacteremia score of 3.0. Among the bacteremic patients, the association between having CAZ-AVI as part of the treatment regimen and 30 day all-cause mortality was significant [odds ratio (OR) and 95% CI = 0.32 (0.12–0.81), p = 0.02] showing a protective effect of the treatment in univariate analysis. After controlling for age, Charlson comorbidity index, and Pitt bacteremia score the association between treatment with CAZ-AVI and 30 day all-cause mortality was no longer significant [AOR and 95% CI = 0.33 (0.10–1.06), p = 0.06]. After adjusting for these predictors, a one-unit increase in Pitt bacteremia score was associated with an increase of 29.0% in the odds of 30 day all-cause mortality [AOR and 95% CI = 1.29 (1.09–1.52), p = 0.004].

Univariate logistic models were constructed to predict the outcome of 30 day all-cause mortality based on demographic and clinical patient characteristics (Table 4). Univariate analysis revealed that a one-unit increase in the Charlson comorbidity index was associated with a 20.0% increase in the odds of dying within 30 days from all causes [OR and 95% CI = 1.20 (1.07–1.33), p = 0.001] and patients with a HAP/VAP infection had an increased odds by a factor of 2.3 of dying within 30 days from all causes [OR and 95%CI = 2.31 (1.15–4.64), p = 0.02]. For patients with a diagnosis of diabetes mellitus at baseline, the odds of dying within 30 days from all-causes increased by a factor of 2.2 times compared to patients without this diagnosis [OR and 95% CI = 2.02 (1.08–3.79), p = 0.03]. All predictor variables with a p < 0.1 were included in the multivariable logistic model looking at the association of clinical characteristics and 30 day all-cause mortality (Tables 4, 5). After adjusting for all predictors with a p < 0.1 from univariate analyses, patients with CRE bacteremia had an increased odds by 281% of dying within 30 days from all causes [AOR and 95% CI = 2.81 (1.26–6.24), p = 0.01]. (Table 5).

**Table 4:** Univariate analysis of patient characteristics associated with 30 day all-cause mortality

Parameter	OR 95% CI	p-value
Age (years)	1.02 (1.01–1.04)	0.01
Charlson comorbidity index	1.20 (1.07–1.33)	0.001
Pitt bacteremia score	1.32 (1.15–1.50)	< 0.0001
APACHE II score	0.99 (0.95–1.02)	0.43
CLABSI	2.74 (1.21–6.22)	0.02
HAP/VAP	2.31 (1.15–4.64)	0.02
Complicated UTI	0.50 (0.22–1.12)	0.09
Complicated intra-abdominal infection	0.37 (0.14–1.03)	0.06
Skin and soft tissue infection	0.75 (0.26–2.17)	0.60
Joint/bone infection	< 0.001 (< 0.001–> 999.99)	0.98
Primary bacteremia (source unidentified)	2.17 (0.75–6.30)	0.15
CRE bacteremia	2.75 (1.45–5.21)	0.002
NDM	1.49 (0.67–3.31)	0.32
OXA48	1.35 (0.68–2.71)	0.39
Received CAZ-AVI	0.75 (0.40–1.40)	0.37
Cardiac disease	1.69 (0.81–3.55)	0.16
Diabetes Mellitus	2.02 (1.08–3.79)	0.03
Non-hematological malignancy	1.30 (0.22–3.37)	0.44
Hematological malignancy	0.86 (0.67–2.49)	0.83
Immunosuppressive medication	1.10 (0.52–2.32)	0.81

*OR* odds ratio, *CI* confidence interval

**Table 5:** Multivariable association of characteristics with 30 day all-cause mortality (n = 189)

Parameter^1^	OR 95% CI	p-value
Age (years)	1.01 (0.99–1.04)	0.29
Charlson comorbidity index	1.08 (0.94–1.24)	0.28
HAP/VAP	1.90 (0.78–4.65)	0.16
Diabetes Mellitus	1.59 (0.77–3.32)	0.21
CRE bacteremia	2.81 (1.26–6.24)	0.01
CLABSI	1.50 (0.49–4.58)	0.48
Complicated intra-abdominal infection	0.45 (0.14–1.51)	0.20
Complicated UTI	0.69 (0.26–1.84)	0.46

*OR* odds ratio, *CI* confidence interval

^1^Categories for characteristics with missing values not included in the table

## Discussion

Our study shows that OXA-48 carbapenemase is the most common in Saudi Arabia followed by NDM carbapenemase. On the other hand, the prevalence of *Klebsiella pneumoniae* carbapenemases (KPC) in the country is quite rare. Mortality in patients with CRE infection is very high which is consistent with other studies published globally [4, 12, 13]. The majority of the CRE in our cohort was Klebsiella pneumonia.

About two-thirds of our patients received carbapenem as part of the treatment regimen during their hospital stay. tThis likely occurred because some of the centers utilized combination therapy for the treatment of CRE infections or patients may have received carbapenem before identification of carbapenem resistance. Around 50% of the cohort received CAZ-AVI for the treatment of CRE infection. CAZ-AVI is a new β-lactam/β-lactamase inhibitor combination with in vitro activity against KPCs and OXA-48 producing Enterobacterales. Several studies showed that CAZ-AVI is effective for the treatment of CRE infection [14–17]. In our study, patients who received CAZ-AVI for CRE were less likely to die compared to those who did not receive it; however, this difference was not statistically significant. There are several reasons for this finding. Approximately one quarter of the isolates tested for susceptibility to CAZ-AVI were resistant to it. In addition, almost 30% of patients in the study cohort were either carrying the NDM gene or an unidentified gene. CAZ-AVI does not have activity against NDM carbapenemases and the efficacy of its combination with aztreonam is less certain when compared to its efficacy as a monotherapy for OXA-48 and KPC [18]. The sample size in this cohort was also not powered to detect the survival benefit of antibiotics intervention. Further examination of patients with CRE infection and bacteremia showed that CAZ-AVI has a protective effect in univariate analysis. Despite being expensive, the use of CAZ-AVI slightly exceeded colistin use which is very nephrotoxic and less effective than CAZ-AVI [15, 16, 19]. Although tigecycline has a black box warning of increased mortality, it is one of the few options available to treat CRE [20], and it was used in about a third of our cohort. Other CRE-active agents were not used because they are not yet available in Saudi Arabia such as eravacycline, plazomicin, meropenem-vaborbactam, and imipenem-cilastatin-relebactam. The latter two are less attractive in our region given their lack of activity against OXA-48, the predominant type of carbapenemase. KPC is not common in the region at this time and can be treated with CAZ-AVI [21].

We identified several predictors for 30-day mortality in the univariate analysis. These included older age, higher comorbidity index, higher pitt bacteremia score, and having diabetes as a comorbidity. These predictors were all consistent with what has been shown in previous studies [22, 23]. HAP/VAP and CRE bacteremia both were associated with a more than two fold increase in mortality. CRE bacteremia has been shown to be associated with a substantial increase in mortality in several studies. After adjusting for potential confounders, CRE bacteremia remained an independent risk factor for mortality.

Our study has several strengths including the prospective design and recruiting patients from several tertiary care hospitals from different geographic locations in Saudi Arabia. Furthermore, we collected information on baseline clinical characteristics and several time-varying covariates. We acknowledge several limitations in our study including the sample size in our cohort precluding identification of strong predictors of mortality and an inability to compare the efficacy of different antibiotic treatments for CRE infection. This was due to the heterogeneity of treatment between centers and to the lack of specific protocols for CRE therapy.

In conclusion, this study improved our knowledge regarding the burden of CRE infection in the region. It also highlights the molecular epidemiology and current therapeutic approach for CRE treatment in Saudi Arabia. This information will inform local and global preventive and therapeutic intervention protocols for CRE infection.

## Data Availability

The datasets used and/or analyzed during the current study are available from the corresponding author on reasonable request.
